# New Metrics for Evaluating Viral Respiratory Pathogenesis

**DOI:** 10.1371/journal.pone.0131451

**Published:** 2015-06-26

**Authors:** Vineet D. Menachery, Lisa E. Gralinski, Ralph S. Baric, Martin T. Ferris

**Affiliations:** 1 Department of Epidemiology, University of North Carolina at Chapel Hill, Chapel Hill, NC, United States of America; 2 Department of Microbiology and Immunology, University of North Carolina at Chapel Hill, Chapel Hill, NC, United States of America; 3 Department of Genetics, University of North Carolina at Chapel Hill, Chapel Hill, NC, United States of America; Deutsches Primatenzentrum GmbH - Leibniz-Institut fur Primatenforschung, GERMANY

## Abstract

Viral pathogenesis studies in mice have relied on markers of severe systemic disease, rather than clinically relevant measures, to evaluate respiratory virus infection; thus confounding connections to human disease. Here, whole-body plethysmography was used to directly measure changes in pulmonary function during two respiratory viral infections. This methodology closely tracked with traditional pathogenesis metrics, distinguished both virus- and dose-specific responses, and identified long-term respiratory changes following both SARS-CoV and Influenza A Virus infection. Together, the work highlights the utility of examining respiratory function following infection in order to fully understand viral pathogenesis.

## Introduction

Modeling infectious disease in small animals has been a major tenet of biomedical research with reports detailing influenza infection of mice in the early 1930s. Mice remain the most widely employed model of respiratory pathogenesis today due to numerous reagents, established protocols, and short reproductive cycles [1]. Mice also allow for evaluation of quantitative aspects of infection including viral replication, host transcriptional responses, and immune infiltration with sufficient numbers to generate statistically robust data, providing information on kinetics and virulence mechanisms otherwise unavailable from either *in vitro* or human cohort studies [2]. Such findings have formed a foundation of knowledge for viral respiratory diseases.

Despite their expansive role, mouse models have several shortcomings in regards to understanding human diseases and viral respiratory infection in particular. Concerns over broad transcriptional dissimilarities [3], the requirement for high viral doses to induce measurable (e.g. weight loss) disease in mice [2], and variation in pathogenicity due to host specific factors [4] have led some to question the utility of small animal models [3]. However, new technologies and approaches including the Collaborative Cross and humanized mice [5–7], provide stronger links between mouse models and human disease. Refined measures of respiratory function (e.g. whole body plethysmography) can provide novel metrics of disease responses that are non-invasively assessed and analyzed in the same animal throughout the course of viral infection. Such approaches present a major opportunity to understand the impact of viral infection on breathing function, develop novel animal models of emerging viral respiratory pathogens, and provide a link to human disease, especially for those pathogens which cause severe morbidity, mortality, and respiratory distress [4,8–10].

In the current work, we utilize infection with mouse adapted Severe Acute Respiratory Syndrome Coronavirus (SARS-CoV) as well as a 2009 Influenza A (IAV) H1N1 isolate (A/California/04/09 (H1N1), henceforth referred to as H1N1-CA04-2009) to model severe acute respiratory disease. In 2003–2004, SARS-CoV emerged from CoVs circulating between bats, civets, and raccoon dogs in open markets causing severe acute respiratory disease with mortality rates exceeding 50% in aged populations [9,11]. Similarly, in the spring of 2009, a novel H1N1 pandemic strain emerged infecting a significant portion of the world’s population and caused substantial morbidity and mortality [12,13]. Importantly, robust mouse models of infection exist for both SARS-CoV and IAV that recapitulate clinical aspect of disease found in humans [4]. Using whole body plethysmography of individual mice in a longitudinal study, our data demonstrates significant changes in a wide variety of respiratory parameters following viral infection. These measurements, Penh, EF50, and Rpef, varied depending on both dose and pathogen throughout this time course. In addition, changes to these metrics correspond with and even precede weight loss changes and lethality, the most traditional measures of pathogenesis. Finally, following the acute phase of infection, changes to these measurements remained, indicating that respiratory virus infection has an impact on breathing function beyond normal acute infection. Together, the results highlight the utility of examining respiratory function via whole body plethysmography within the context of acute respiratory infection.

## Materials and Methods

### Cells and Virus

Viral titration and propagation of SARS-CoV and influenza A viruses were preformed in VeroE6 and MDCK cells, respectively, using standard methods. Wild-type (WT) mouse adapted infectious clone (ic) SARS-CoV was derived from the Baric laboratory’s ic constructs [10]. Influenza A/California/04/2009 (H1N1-09) were derived from a plasmid-based reverse-genetic system and subsequently amplified in MDCK cells [14].

### Mice

20 week old C57BL/6J animals were ordered from the Jackson Labs (Bar Harbor, ME). Animals were brought into a biosafety lab, level 3 and allowed to acclimate for 1 week prior to infection. For infection, mice were anesthetized with a mixture of ketamine and xylazine and infected intranasally when challenged with 50ul of phosphate-buffered saline (PBS) or diluted virus (SARS-CoV MA15 or IAV) with three to four mice per infection group per dose as described in the figure legends. As per animal protocol, animals were monitored daily for clinical signs of disease (hunching, ruffled fur, reduced activity) for the duration of the experiment. Weight loss was monitored daily for the first 10 days after which, weight monitoring continued until the animals recovered to their initial starting weight or displayed three continuous days of weight gain. All mice losing greater than 20% of their starting body weight were ground fed and further monitored multiple times per day as long as they were under the 20% cutoff. Mice losing greater than 30% of their starting body weight were immediately sacrificed as per protocol. Any mouse deemed to be moribund or unlikely to recover were also humanly sacrificed at the discretion of the researcher. Euthanasia was preformed via isoflurane overdose and confirmation of death by cervical dislocation. All mouse studies were performed at the University of North Carolina (Animal Welfare Assurance #A3410-01) using protocols approved by the UNC Institutional Animal Care and Use Committee (IACUC).

### Infection

Animals were anesthetized with a mixture of ketamine/xylezine. Virus inoculum was diluted to the appropriate concentration in phosphate buffered saline, and was introduced intranasally in a volume of 50 microliters. The dose curve for SARS-CoV infection was 10^5^, 10^4^ 10^3^ plaque forming units per 50 microliters; for the expanded time course, the infectious dose for H1N1-09 was 5x10^3^ and SARS-CoV MA15 was 10^4^ plaque forming units per 50 microliters.

### Plethysmography

Animals were individually introduced to a plethysmography chamber (Buxco Systems, Wilmington, NC) located within the biological safety cabinet and acclimated for 30 minutes. Following acclimation, animals were actively measured for a 5 minute period. Animals were introduced into the chamber once per day, with their first introduction into the plethysmography chamber immediately prior to infection, and thereafter at indicated time points post infection. Animals were randomly assigned to a given chamber each day.

### Data and Statistical Analysis

All statistical and data analysis were conducted within the R environment (cran.r-project.org). The package doBy was used for data manipulation, MASS was used for data transformations for normality, lme4 for the mixed-effect statistical analyses, and ggplot2 for data visualization.

**Fig 1:**
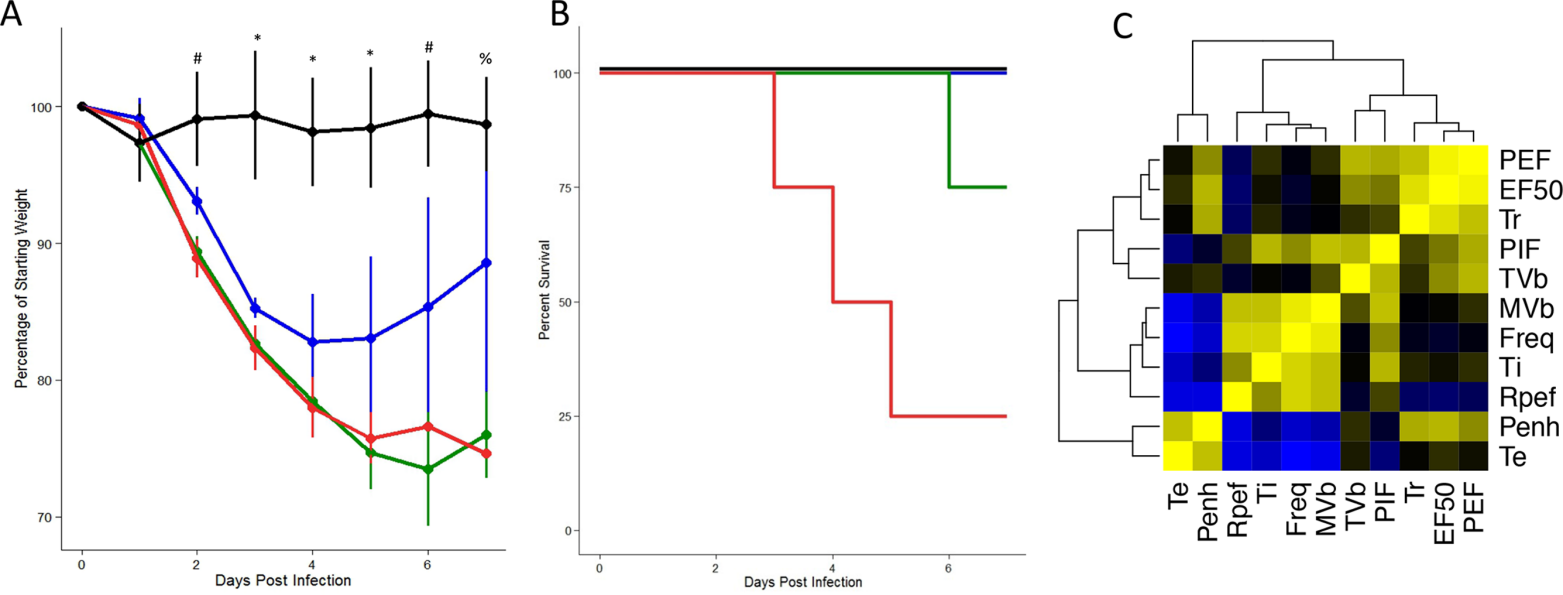
Dose-dependent respiratory stress following SARS-CoV infection. Four C57BL/6J animals per group were either mock-infected (black) or infected with increasing doses of SARS-CoV (10^3, Blue; 10^4, Green; 10^5, Red). Weight loss (A), Mortality (B), and Respiratory parameters (Sup. Data) were measured through 7 days post infection. Whether there was a significant effect of treatment on weight loss (A) was determined via partial F-test. Following significance assessment, those treatment groups different from each-other were assessed by Tukey’s HSD post-hoc analysis. All such differences are denoted at a p<0.05 level, and are marked as follows: * = mock different from all infected, # = mock different from all infected; 10^3 different from 10^4 and 10^5, % = mock different from 10^4 and 10^5 doses. C) UPGMA (**U**nweighted **P**air **G**roup **M**ethod with **A**rithmetic Mean)-Clustered correlation matrix describing the relationship between various plethysmographic outputs. For each pair of transformed phenotypes, the correlation between these phenotypes was calculated. The color of each cell relates to the strength of correlation (ranging from -1 at light blue, no correlation being black, and a +1 correlation being bright yellow). In this way strong positive and negative relationships, as well as clusters of tightly related phenotypes could be identified across the range of SARS-CoV dose responses.

During the 5-minute measurement period within the plethysmograph, the plethysmograph continually measures respiratory responses, and outputs these data as 150 2-second summaries of 11 various respiratory measures (shown in Fig 1C and S1 Table), as well as several data quality controal metrics. Output data were examined and transformed to normality using the Box-Cox approach.

Within a day’s measurement period, the 150 sequential measures provide increased information about the animal’s respiratory function as well as the causal relationships between respiratory metrics, over just examining the per-day animal’s average measure of these data. At the same time, uncareful utilization of these sequential measures can result in inflated statistical significance. We therefore settled on a partial f-test framework (analogous to model selection approaches). Specifically, we first fit a mixed effect null model:

H0:Respiratorymeasure=Day+(1|Subject)

Where Day is a continuous independent fixed effect, and Subject is a random effect. This approach allows for the unique animal-to-animal physiology to be accounted for, as well as for daily variation in measures to be accounted for. We then contrast the null model with our test model:

H1:Respiratorymeasure=Day+Treatment+(1|Subject)

This contrasting test allows us to specifically determine a standard p-value assessing the significance of treatment effects (e.g. viral pathogen, viral dose) significantly increases the fit (reduces the error estimate) of our statistical model that already accounts for daily and animal-specific effects.

If a significant overall effect of treatment on respiratory responses was seen throughout the course of infection, we then used a similar framework within days to determine at which time points post infection there were bonferonni corrected (p<0.0071) significant effects of treatments. Post-hoc comparisons were then made using Tukey’s HSD approach to identify those treatment groups (e.g. mock versus flu-infected versus SARS-infected differences) specifically differing from each other at given timepoints.

### Ethics Statement

The present study was carried out in accordance with the recommendations for care and use of animals by the Office of Laboratory Animal Welfare, National Institutes of Health. The Institutional Animal Care and Use Committee (IACUC) of The University of North Carolina at Chapel Hill (UNC; permit A-3410-01) approved the animal study protocol (IACUC 13–026) followed here.

## Results

### Dose-dependent respiratory response to SARS-CoV

Our studies built upon a well established infection model of SARS-CoV, utilizing a mouse adapted virus (MA15) in 20-wk old C57BL/6J mice, which produces a range of dose-dependent disease including robust viral titers in the lung, weight loss, histological damage, and mortality [15]. Initial results from these studies confirm previously reported dose-dependent changes in weight loss (Fig 1A) and survival (Fig 1B). Concurrently, we also utilized whole body plethysmography (Buxco) to measure respiratory parameters in the context of infection over the seven-day time course (S1 Table). These plethysmography outputs, which include a variety of primary respiratory measures (e.g. frequency, tidal volume and inspiratory/expiratory flow rates) as well as derived respiratory measures (e.g. airflow resistance), which are summarized (S2 Table) and provide novel metrics to evaluate the impact of viral infection on respiratory function. Importantly, both the complexity and correlation structure between these respiratory measures provides extensive depth in describing pathogenesis following infection (Fig 1C, S1 Table). For these studies, we focused on three specific metrics that change in the context of infection: Penh, EF50, and Rpef.

### Penh

**Fig 2:**
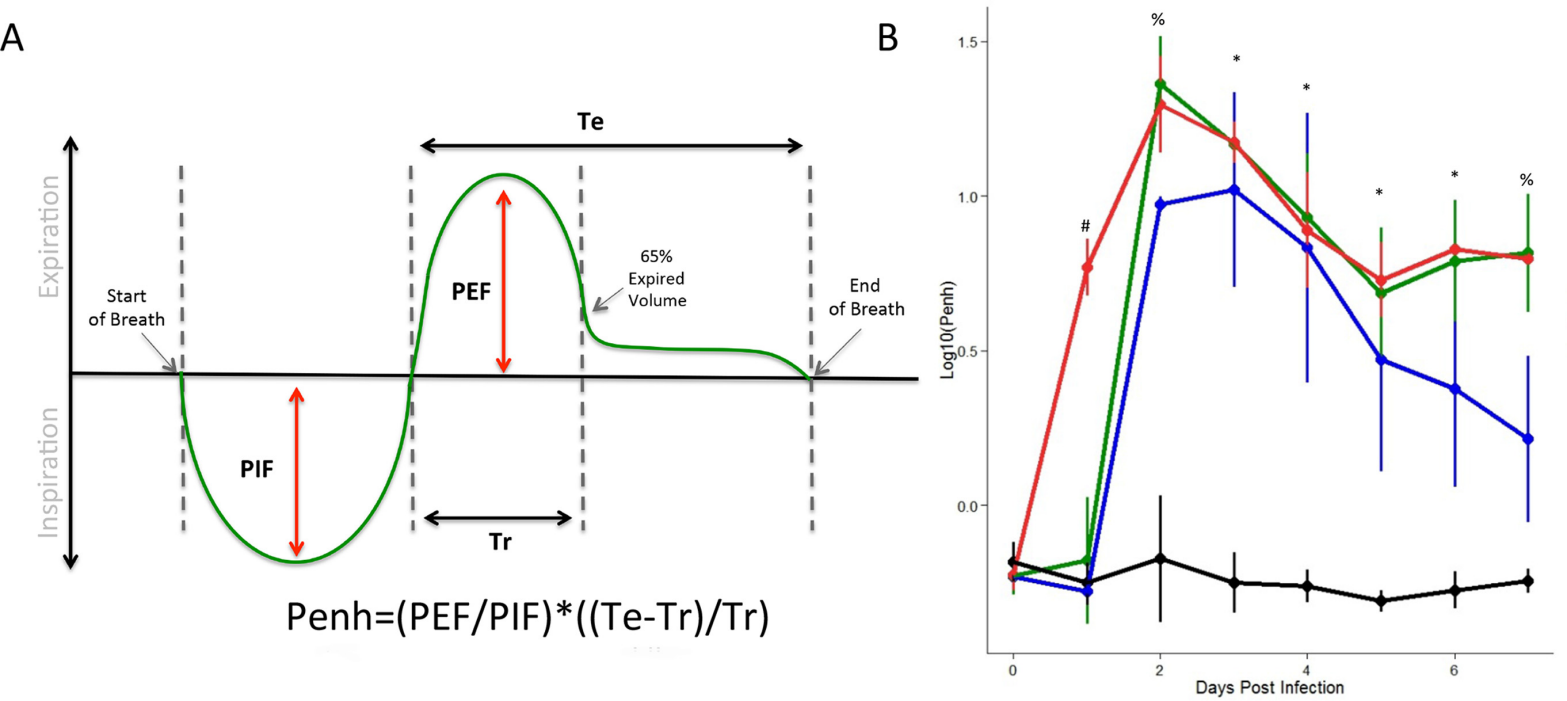
Penh shows increased sensitivity in dose-dependent responses following SARS-CoV infection. Penh is a classically used, and derived measure of respiratory distress. (A) Penh is derived by assessing several measures of the respiratory response curve (peak expiratory flow of breath (PEF), peak inspiratory flow of breath (PIF), time of expiratory portion of breath (Te) and time required to exhale 65% of breath volume (Tr) (B) Following SARS-CoV infection of C57BL/6J animals, we identified significant differences in Penh across a range of doses relative to mock animals (black = mock, blue = 10^3 SARS, green = 10^4, red = 10^5; four animals per group). Within a time point, letters indicate groups that are NOT significantly different from each other. Significant effects of treatment on Penh was determined via partial F-test. Following significance assessment, those treatment groups different from each-other were assessed by Tukey’s HSD post-hoc analysis. All such differences are denoted at a p<0.05 level, and are marked as follows: * = mock different from all infected, # = 10^5 dose different from all others, % = mock different from all doses, 10^3 different from 10^4 and 10^5 doses.

The first respiratory metric that we focused on is enhanced pause (Penh), a unit-less index of calculated airway function [16]. The equation for Penh (Fig 2A) takes into account four breathing parameters including peak expiratory flow of breath (PEF), peak inspiratory flow of breath (PIF), time of expiratory portion of breath (Te), and time required to exhale 65% of breath volume (Tr). While a novel metric for *in vivo* infection, penH derived from mouse whole body plethysmography is not without controversy in the asthma field. Supporters suggests that penH serves as an indirect measure of airway resistance while critics argue it fails to measure airway constriction and at best provides non-specific assessment of breathing patterns [16–18]. In this study, SARS-CoV infection resulted in a dose responsive change in Penh values (Fig 2B). While mock-infected animals maintained baseline levels, SARS-CoV infection at each dose produced increased Penh values during the early portion of the infection time course. While PEF, PIF and Tr values showed variability, changes in Penh were primarily driven by increases in Te during SARS-CoV infection (Fig 1C). Importantly, the increases in Penh corresponded with, and in the case of the 10^5^ dose, preceded changes in weight loss (Fig 1A), providing an additional metric for the onset of pathogenesis. Notably, our study shows high (values greater than 10) and sustained values of Penh, which exceed previously reported values in both viral and asthma airway responsiveness studies, which typically result in transitive levels of Penh in the 1–3 range with mock animals typically showing values <<1 [19–21]. As the time course continued, Penh values also began to decline in a dose-dependent manner, but remained significantly elevated at the termination of the experiment. Despite the surrounding controversy, Penh provides a novel respiratory metric of pathogenesis in the context of SARS-CoV infection that is highly discriminating of variation in viral doses, and also corresponds with and/or precedes changes in weight loss.

### EF50

**Fig 3:**
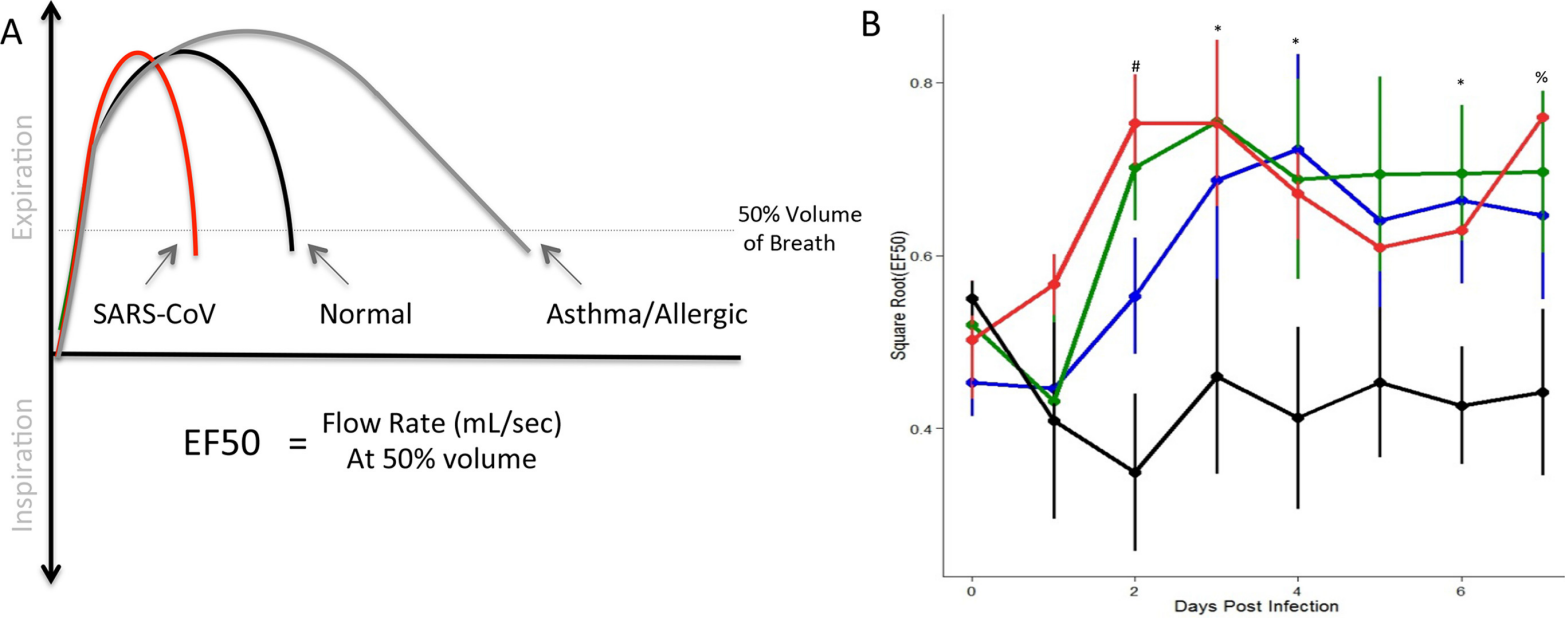
Mid-tidal expiratory flow (EF50) demonstrated dose-dependent increase following SARS-CoV infection. (A) Measuring the flow rate at which 50% of the tidal volume has been expelled, EF50 provides information about the early portion of the respiratory curve and has demonstrated notable differences between normal (black), asthmatic/allergic (Gray), and viral infection (red). (B) Following SARS-CoV infection of C57BL/6J animals, we identified significant differences in EF50 across a range of doses relative to mock animals (black = mock, blue = 10^3 SARS, green = 10^4, red = 10^5; four animals per group). Significant effects of treatment on EF50 was determined via partial F-test. Following significance assessment, those treatment groups different from each-other were assessed by Tukey’s HSD post-hoc analysis. All such differences are denoted at a p<0.05 level, and are marked as follows: * = mock different from all infected, # = mock different from all infected; 10^3 different from 10^4 and 10^5, % = mock different from 10^4 and 10^5 doses.

The second respiratory metric that we focused on is mid-tidal expiratory flow (EF50). EF50 values represent the flow rate at which 50% of the tidal volume of an individual breath has been expelled, measured in milliliters per second [22]. While EF50 bears some similarities with forced expiratory flow 50% (FEF50), a standard measure in human spirometry [23], differences in the wave form and magnitude between natural and forced breaths make comparisons difficult. Despite these differences both measures can provide insights into the early portion of the breathing curve. Typically, *in vivo* asthma studies examining respiratory function have observed reduced level of EF50, reporting percent reduction relative to baseline [22] [24]. The resulting breath curve shifts to the right, as the reduced flow rate requires more time to exhale 50% of the volume (Fig 3A). However, EF50 values increased robustly during SARS-CoV infection compared to mock with maximum differences occurring between 2 and 4 days post infection (DPI) (Fig 3B). These kinetics varied based on condition, with 10^4^ and 10^5^ doses inducing a more rapid increase and maintenance of high EF50 levels throughout the time course of infection. These data indicate that during SARS-CoV infection, the breath curves shifts to the left, more rapidly exhaling breath to 50% volume (Fig 3A). Together, the results represent a striking contrast with prior studies examining airway function in the context of allergic responses [24]. In addition, detectable differences between each of the SARS-CoV infection groups indicate dose dependent kinetics that may distinguish disease severity prior to other pathogenic markers like weight loss and lethality.

### Rpef

**Fig 4:**
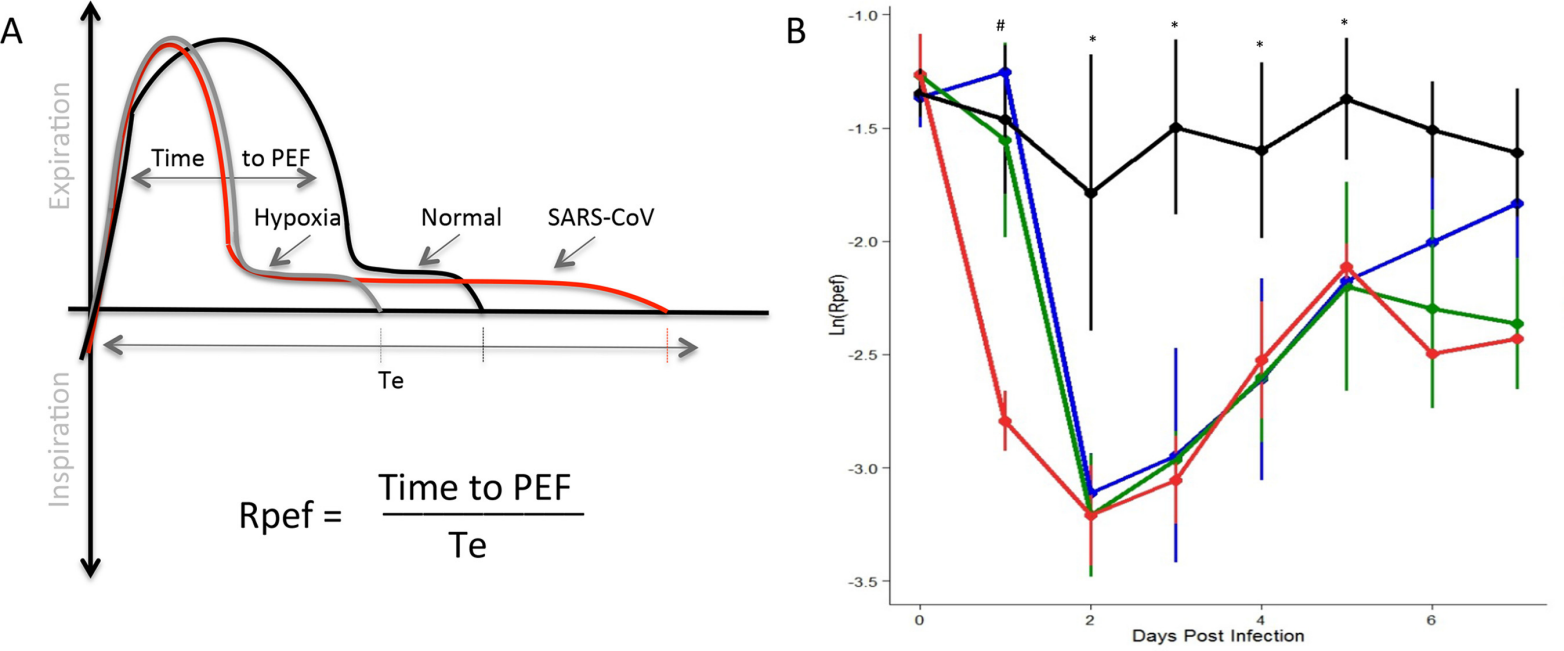
The shape of the exhalatory flow curve (Rpef) indicated changes following infection with SARS-CoV. (A) Rpef measures the ratio of time to peak expiratory follow (PEF) relative to the total expiratory time. For both hypoxia (gray) and SARS-CoV infection (red), the time to PEF decreases relative to normal (black). However, the length of breath expands following SARS-CoV infection, causing significant drop in Rpef values relative to baseline. (B) Following SARS-CoV infection of C57BL/6J animals, we identified significant differences in Rpef across a range of doses relative to mock animals (black = mock, blue = 10^3 SARS, green = 10^4, red = 10^5; four animals per group). Significant effects of treatment on Rpef was determined via partial F-test. Following significance assessment, those treatment groups different from each-other were assessed by Tukey’s HSD post-hoc analysis. All such differences are denoted at a p<0.05 level, and are marked as follows: * = mock different from all infected, # = 10^5 dose different from all others.

The final respiratory metric that we focused on was Rpef, the ratio of time to peak expiratory flow (PEF) relative to total expiratory time (Te). Similar to both Penh and EF50, Rpef provides a calculated index of the breath curve combining the time needed to reach maximum expiratory flow divided by the total length of the breath (Fig 4A). While less utilized than the previous two metrics, Rpef values have been shown to increase in the context of hypoxia *in vivo* [25]. However, in our studies, SARS-CoV infection results in a robust decline of Rpef relative to mock controls which remain static over the time course (Fig 4B). Similar to the kinetics of Penh, reduction in Rpef values corresponded with (10^3^, 10^4^ dose) or preceded (10^5^) changes in weight loss. As the time course continued, Rpef recovery was equivalent at each dose through D5, but diverged thereafter. Examining the underlying respiratory parameters revealed a more rapid time to PEF following SARS-CoV infection, consistent with previous studies of hypoxia. However, the increase in time to PEF also corresponded with a longer breath (Te), resulting in an increased denominator in the ratio and driving significant reductions in Rpef values. Importantly, the kinetics of the Rpef response illustrated a dose dependent impact of SARS-CoV infection and provides a third, clinically relevant respiratory metric for evaluating pathogenesis *in vivo*.

### Respiratory function of SARS-CoV versus Influenza A virus

**Fig 5:**
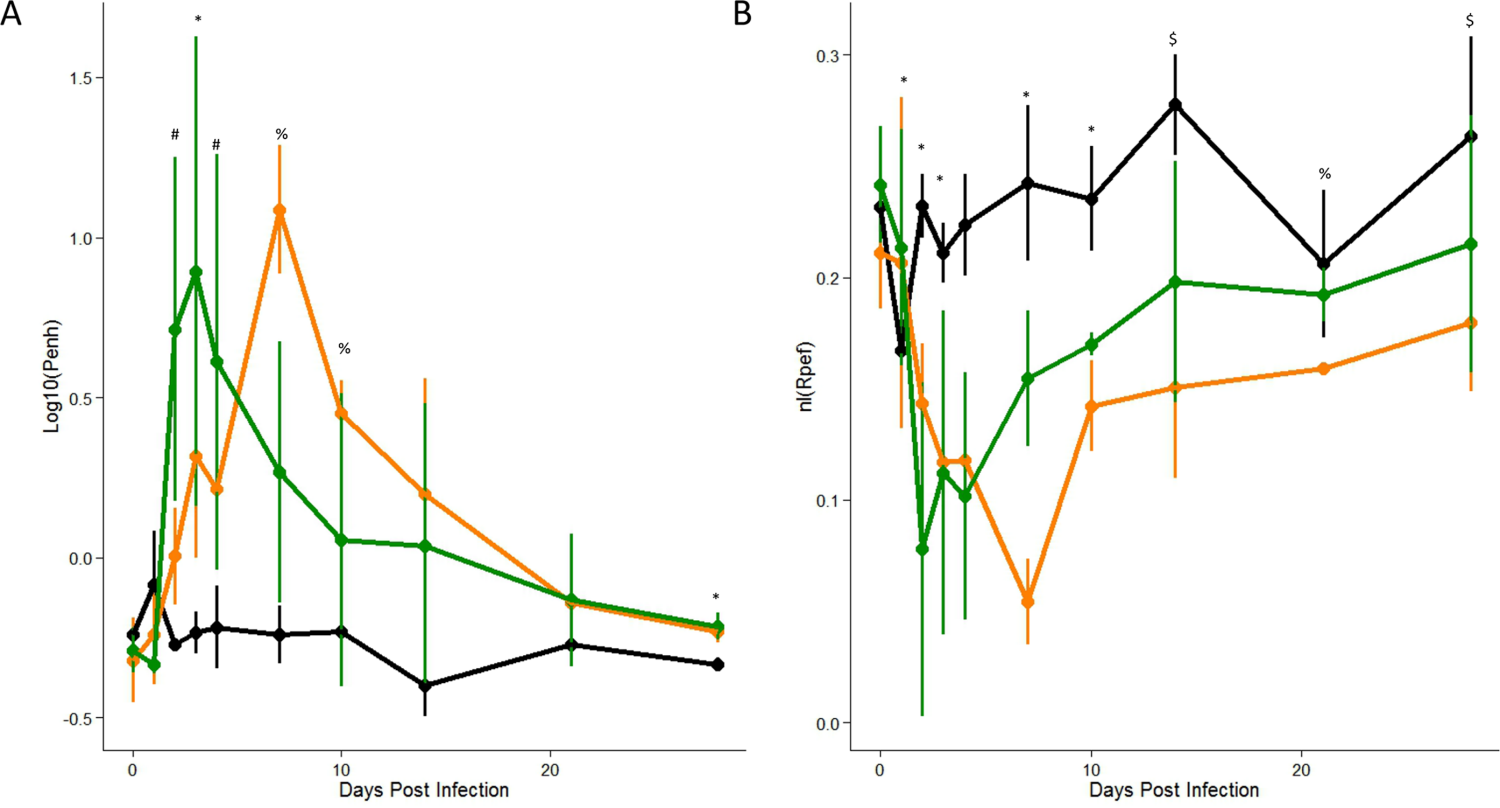
Differential responses to two respiratory pathogens. C57BL/6J mice were mock-infected (Black, n = 3) or infected with 10^4 of SARS-CoV (Green, n = 4) or 10^4 IAV-H1N1-09 (Orange, n = 4), and Airflow resistance, penH (A) or the shape of the expiratory force curve, Rpef (B) were measured through 28 days post infection. Significant effects of treatment on respiratory responses were determined via partial F-test. Following significance assessment, those treatment groups different from each-other were assessed by Tukey’s HSD post-hoc analysis. All such differences are denoted at a p<0.05 level, and are marked as follows: * = mock different from all infected, # = SARS different from mock and flu; %Flu different from SARS and mock, $ = Flu different from mock.

In order to validate as well as expand on these findings, we conducted additional experiments that explored differences between respiratory pathogens as well as the long-term impact on breathing function. Using semi-lethal doses, we compared SARS-CoV (10^4^) to a human isolate of 2009 Influenza A virus, (IAV-H1N1-09) (10^4^ pfu) over a 28 day time course. Based on previous studies, weight loss for SARS-CoV occurs primarily between D2 and D5 post infection (Fig 1); in contrast, IAV-H1N1-09 weight loss is delayed with major weight loss occurring between D4 and D8 post infection (S3 Table) [4]. In regards to respiratory metrics, both viruses show similar overall magnitudes in their Penh and Rpef responses (Fig 5A and 5B, S4 Table). Similarly, EF50 showed evidence of enhancement in both SARS and flu infection relative to mock animals (S5 Table). However, the kinetics of these response curves varied, with SARS-CoV showing earlier changes and resolution compared to IAV-H1N1-09. As in our initial experiment, for both viruses, changes in respiratory physiology corresponded with weight loss and confirmed previously observed pathogenesis trends in regards to histopathology scoring (S6 Table). In contrast to prior studies, we were also able to identify significant long-term changes in both respiratory metrics following infection despite recovery from infection-induced weight loss. As far as 28 days post infection, both Penh and Rpef levels remained significantly different from those observed in mock animals. These results suggested that despite the absence of other metrics of pathogenesis, breathing function requires additional recovery time, may not return to baseline levels following respiratory virus infection, and might provide insight into chronic or secondary-infection responses attributed to both SARS and IAV [26].

## Discussion

In contrast to traditional measures of respiratory virus pathogenesis, examination of breathing function represent a novel and clinically relevant metric of disease following infection. Our studies illustrated significant changes to several respiratory parameters including penH, EF50, and rPEF following both SARS-CoV and IAV-H1N1-09 infection. Importantly, these respiratory measures track with, and at times, preceded more traditional measures of disease pathogenesis including weight loss and lethality, but also appear independent of viral load. Observation of these respiratory metrics provides increased depth to pathogenesis studies. In addition, the impact on respiratory function extends beyond acute infection and this extension is not currently captured by more traditional metrics of pathogenesis. Together, examination of these respiratory measures provided novel insight into the onset, severity, and kinetics of disease responses that were robust across two viral pathogens, improved ties to measures of human respiratory function, and gave insights into the functional outcomes of virus infections.

As with many experimental studies, interpretation of these respiratory metrics is complicated by having only indirect parallels in human pulmonary function testing. While human tests primarily utilized measures of forced breath, similar metrics cannot be applied to *in vivo* studies of mice. Despite these differences, important insights into infection can be ascertained. The time length of breath (Te) increased during the peak time of disease following both SARS-CoV and IAV infection. This change in Te drove differences observed between infected and mock treated animals, and was captured in both Penh and Rpef (Fig 1c). Surprisingly, despite the increased length of breath, the flow rate of expiration during the early part of the breath also increased, as captured in the highly correlated PEF and EF50 (Fig 1c). Coupled with a faster time to reach peak flow rate (in Rpef), the data suggested that viral infection results in a rapid exhalation of the majority the air volume. However, the remainder of the volume takes longer to release, analogous to a wheeze observed in humans. Applying traditional descriptions of human airway disease, these metrics appear to overlap elements of both restrictive and obstructive airway disease patterns. Cross-referencing observations from SARS-CoV infection: damage and debris from infected lung epithelia coupled with infiltration of immune populations have been shown to enhanced fibrinosis and wound healing responses in vivo [9,15]. The expected impact on the lung would be decreased pulmonary compliance (i.e. more stiff), possibly resulting in a more rapid flow rate during the early portion of breath, and a reduced rate toward the end, elongating the time of breath. Therefore, the respiratory metrics measured in these studies are likely consistent with observed pathogenesis in humans, and illustrate the utility of following these values as measures of pathogenesis.

While EF50 and Rpef are relatively novel respiratory metrics [24,25], enhanced pause (Penh) has a longer and more controversial history. Within the asthma field, the use of Penh as a marker of resistance has been debated with support both for and against its use [16–18]. In several ways, our results and interpretation seek to be independent of such controversy. While typical asthma studies use methacholine or other allergens to induce an immediate and acute airway constriction [17], our studies required no priming of the animal beyond initial infection. The penH values we observed tracked with, and at times preceded, more classical measures of pathogenesis and disease (Fig 2B); most importantly, the Penh peak closely corresponds to the peak levels of viral replication, airway denudation, and debris for both SARS-CoV and IAV (S6 Table) [15,27]. Importantly, these airflow changes were maintained for several days rather than the minutes observed in asthma studies. Finally, we observed that the kinetics of Penh varied based on the pathogen, indicating changes were not due to an artifact of the whole body plethysmography system. A modest drop in penH over the time course tracks with clearing of the airway, but also with increased inflammation and cellular infiltration. Together, the data indicated that Penh corresponds to histological markers of pathogenesis *in vivo*. In addition, the results suggested that for infectious diseases, airway denudation and debris in the context of high viral replication likely provide a major mechanical contribution to airway resistance, contrasting the role of airway constriction on airway resistance in asthma [15]. Future studies directly contrasting our unrestrained plethysmography approaches with more direct measures of airway resistance (e.g. forced ventilation [28]) may provide useful insight into the relative roles of constriction and tissue damage on respiratory distress following viral infection. Regardless of the ultimate functional mechanism, penH clearly provides a useful characterization of altered respiratory function and differentiates pathogeneicity following infection in vivo.

The addition of these respiratory pathogenesis markers provides an opportunity to further characterize infection, revolutionize our understanding of disease, and develop novel therapuetics for the treatment. By capturing these respiratory metrics, infection with new and novel strains can be expanded beyond systemic markers like weight loss and lethality. This depth permits further classification of attenuation and provides the opportunities to identify the nature and pathologic functions that disntiguishing these strains. For example, previous studies using different plethysmography outputs with influenza H3N2 infected animals demonstrated both dose and strain dependent differences not captured by weight loss alone [29]; similar plethysmography studies provided additional metrics to distinguish lethal and sublethal disease phenotypes following H1N1 [28]. Both influenza results are consistent with data for SARS-CoV and expand upon the single dose influeza studies described in this manuscript. Together, the results demonstrated the utility in employing plethysmography in baseline pathogenesis studies. In addition, whole body plethysmography can be utilized to explore the impact of attenuating mutations in vivo. Using whole body plethysmography, studies demonstrated attenuation and restoration of a 2’O methyltransferase mutant of SARS-CoV in terms of respiratory function in addition to deficits in weight loss and viral titer [30]. These findings spurred application of this mutant as a live attenuated vaccine platform at least partially due to the minimal disruption of respiratory function [31]. Similar approaches with other novel and emerging viruses provide the opportunity to fully examine respiratory function and determine pathogensis potential.

In addition to further characterization of physiological mechanisms underlying severity of viral infection, these respiratory metrics may also provide insights into the treatment, resolution, and long-term physiological impacts of infection. While most drug targets focus on reducing viral replication/infection, modulating the host response may provide a better means to alleviate the acute and downstream impact of infection. However, traditional metrics of pathogenesis like weight loss and viral titer are not the only appropriate measures for this type of evaluation. Instead, respiratory metrics like Penh, EF50, and Rpef provide an opportunity to evaluate treatments that target host pathways and the impact on overall infection. For example, immune pathology has been a hallmark of pathogenic respiratory virus disease. A number of treatment options that target host pathways that include complement, urokinase pathways, and other wound repair processes may reduce immune pathology, independent of viral replication, improving lung function [15]. Similarly, these measures can be used to evlaute the kinetics of treatment, identifying ideal windows for application based on respiratory outcomes. Importantly, examining Penh, EF50, and Rpef can lead to treatment options that look beyond acute infection and improve long-term lung function, especially important in highly vulnerable elderly populations. While small, significant changes are noted in long-term baseline functions of Penh and Rpef (Fig 5); these minor changes in young mice are likely to expand in aged animals and may contribute to age related susceptiblity observed both in mice and humans [32]. Together, these results provide metrics that respresent an opportunity to understand and improve treatment of disease following respiratory virus infection.

Overall, the current work highlights the utility of examining physiological metrics of infection, and specifically whole body plethysmography in the context of virulent respiratory virus infection. For both SARS-CoV and IAV, distinct respiratory changes occur following infection and these new metrics provide increased depth of understanding in regards to pathogenesis. While at this point, the underlying functional mechanisms remain unclear, the value of Penh, EF50, and Rpef are illustrated by their concordance and increased sensitivity as compared to traditional markers of pathogenesis. Importantly, respiratory metrics also demonstrate the long-term impact of infection on lung function that is currently not captured by other pathogenesis markers. Finally, the examination of lung function in mice provides an important link to human respiratory disease which may facilitate treatment options that target host processes in order to improve respiration during infection. Together, the results highlight the utility and potential of examining whole body plethysmography within the context of acute respiratory virus infection *in vivo*.

## Supporting Information

S1 Table Daily means of respiratory phenotypes across SARS-CoV dose response. (PDF)

S2 Table Significant Differences in Respiratory Function in SARS dose responses. (PDF)

S3 Table Weight loss following IAV and Mock infection. (PDF)

S4 Table Daily means of respiratory phenotypes across two respiratory pathogens. (PDF)

S5 Table Significant Differences in Respiratory Function in specific virus infection. (PDF)

S6 Table Histopathology Scoring of Lung Disease. (PDF)
