# Supplementary material for: New Metrics for Evaluating Viral Respiratory Pathogenesis
Source: PLoS One. 2015 Jun 26;10(6):e0131451. doi: 10.1371/journal.pone.0131451 (PMC4482571; doi:10.1371/journal.pone.0131451)
Supplement: S1 Table — (PDF) [file pone.0131451.s001.pdf]

Supporting Information Table S1: Daily means of respiratory phenotypes across SARS-CoV dose response

Day	Treatment	Sqrt(Freq)	Log10(TVb)	Log10(MVb)	Log10(Penh)	LN(Rpef)	Sqrt(PIF)	Sqrt(PEF)	1/Ti	Log10(Te)	Sqrt(EF50)	1/Tr
0	Mock	22.75	-0.61	2.1	-0.18	-1.35	3.14	2.47	24.48	-1.05	0.55	22.55
	10^3	21.17	-0.7	1.94	-0.23	-1.36	2.8	2.05	23.57	-0.95	0.45	18.08
	10^4	22.38	-0.64	2.06	-0.23	-1.27	3.05	2.32	24.28	-1	0.52	20.11
	10^5	21.32	-0.63	2.02	-0.23	-1.26	2.97	2.46	22.6	-0.95	0.5	17.77
1	Mock	18.46	-0.68	1.82	-0.25	-1.46	2.54	1.89	18.78	-0.81	0.41	14.25
	10^3	21.04	-0.7	1.93	-0.28	-1.25	2.76	2	23.34	-0.93	0.45	16.95
	10^4	19.2	-0.68	1.87	-0.18	-1.55	2.64	1.97	19.77	-0.84	0.43	15.64
	10^5	13.88	-0.57	1.69	0.77	-2.79	2.74	2.44	17.05	-0.48	0.57	24.07
2	Mock	15.9	-0.7	1.67	-0.17	-1.79	2.24	1.73	15.38	-0.69	0.25	11.68
	10^3	11.63	-0.54	1.58	0.97	-3.11	2.57	2.54	13.57	-0.35	0.55	21.75
	10^4	10.19	-0.58	1.42	1.36	-3.21	2.37	2.69	12.71	-0.2	.7	28.17
	10^5	10.59	-0.55	1.48	1.3	-3.21	2.64	2.83	14.79	-0.25	0.75	30.72
3	Mock	19.97	-0.63	1.95	-0.25	-1.5	2.84	2.2	20.98	-0.9	0.46	17.35
	10^3	12.67	-0.55	1.62	1.02	-2.95	2.74	2.78	16.05	-0.43	0.69	29.6
	10^4	11.71	-0.56	1.56	1.17	-2.96	2.7	2.86	15.72	-0.69	0.35	11.68
	10^5	11.4	-0.58	1.52	1.17	-3.05	2.81	2.86	16.93	-0.34	0.75	33.12
4	Mock	18.54	-0.65	1.86	-0.26	-1.6	2.6	2	18.58	-0.82	0.41	14.88
	10^3	14.84	-0.54	1.77	0.83	-2.61	2.92	2.87	18.07	-0.6	0.72	32.27
	10^4	13.94	-0.67	1.6	0.93	-2.6	2.51	2.55	17.01	-0.56	0.69	33.01
	10^5	14.48	-0.71	1.6	0.89	-2.52	2.49	2.47	18.14	-0.6	0.67	34.58
5	Mock	20.17	-0.66	1.92	-0.31	-1.37	2.72	2.07	21.19	-0.91	0.45	16.94
	10^3	17.72	-0.61	1.87	0.47	-2.17	2.84	2.56	20.02	-0.78	0.64	29.41
	10^4	16.79	-0.68	1.76	0.69	-2.2	2.66	2.57	19.59	-0.74	0.69	34.63
	10^5	16	-0.77	1.63	0.73	-2.11	2.29	2.28	17.2	-0.71	0.61	32.67
6	Mock	19.17	-0.64	1.9	-0.27	-1.51	2.75	2.06	20.25	-0.91	0.43	16.18
	10^3	19.08	-0.57	1.98	0.37	-2	3.15	2.71	21.9	-0.84	0.66	30.53
	10^4	15.78	-0.67	1.72	0.79	-2.3	2.62	2.62	18.63	-0.69	0.69	35.07
	10^5	14.26	-0.7	1.6	0.83	-2.49	2.49	2.37	17.63	-0.57	0.63	31.42
7	Mock	19.21	-0.62	1.91	-0.24	-1.61	2.83	2.14	20.58	-0.84	0.44	16.44
	10^3	20.13	-0.54	2.05	0.21	-1.83	3.28	2.7	22.39	-0.91	0.64	28.9
	10^4	15.91	-0.68	1.71	0.82	-2.36	2.66	2.62	19.79	-0.68	0.7	37.07
	10^5	15.17	-0.56	1.79	0.8	-2.43	3.08	2.88	19.85	-0.63	0.76	34.41
