# Supplementary material for: New Metrics for Evaluating Viral Respiratory Pathogenesis
Source: PLoS One. 2015 Jun 26;10(6):e0131451. doi: 10.1371/journal.pone.0131451 (PMC4482571; doi:10.1371/journal.pone.0131451)
Supplement: S2 Table — (PDF) [file pone.0131451.s002.pdf]

Supporting Information Table S2: Significant Differences in Respiratory Function in SARS dose responses

Phenotype	Differences	Timepoints
Sqrt(Freq)	Infected vs. Mock	D2, D3
	Dose Response	D2
Log_{10}(TVb)	Infected vs. Mock	D2
	Dose Response	N/A
Log_{10}(MVb)	Infected vs. Mock	D3
	Dose Response	D6
Log_{10}(Penh)	Infected vs. Mock	D1-D7
	Dose Response	D1, D2, D7
LN(Rpef)	Infected vs. Mock	D1-D5
	Dose Response	D1, D5
Sqrt(PIF)	Infected vs. Mock	N/A
	Dose Response	D6
Sqrt(PEF)	Infected vs. Mock	D2
	Dose Response	N/A
1/Ti	Infected vs. Mock	N/A
	Dose Response	N/A
Log_{10}(Te)	Infected vs. Mock	D2, D3
	Dose Response	N/A
Sqrt(EF50)	Infected vs. Mock	D2-D4, D6, D7
	Dose Response	D2, D6, D7
1/Tr	Infected vs. Mock	D2-D7
	Dose Response	D2

For each phenotype at each day, a partial F-test was used to identify those days on which treatment had a significant effect on respiratory phenotypes. We then used Tukey's HSD to identify whether those significant effects of treatment were due to respiratory differences between mock and infected animals, or between the different dose categories. Phenotypes: Freq=frequency, TVb=Tidal Volume, MBv=Minute Volume, penH=Enhanced Pause, rPEF=ratio of time to peak expiratory flow, PIFb=peak inspiration, PEFb=peak expiration, Ti=inspiratory time (milliseconds), Te=expiratory time (milliseconds), EF50=Mid-tidal expiratory flow, Tr=relaxation time.
