# Supplementary material for: New Metrics for Evaluating Viral Respiratory Pathogenesis
Source: PLoS One. 2015 Jun 26;10(6):e0131451. doi: 10.1371/journal.pone.0131451 (PMC4482571; doi:10.1371/journal.pone.0131451)
Supplement: S3 Table — (PDF) [file pone.0131451.s003.pdf]

Supporting Information Table S3: Weight loss following IAV and Mock infection

Cage/Mouse #		Virus	Day 0	Day 1	Day 2	Day 3	Day 4	Day 5	Day 6	Day 7	Day 8	Day 9	Day 10	Day 11	Day 12	Day 13	Day 14	Day 15	Day 16	Day 17
Flu	L-1	IAV	20.6	18.9	19.6	19.1	17.1	17.1	16.7	15.6	14.9	15	15.4	16.3	17.6	18.6	18.5	18.7	19.5	19.7
Flu	L-2	IAV	22.3	21.5	22.3	20.9	19.2	19.2	18.3	17.2	16.3	16.3	16.4	16.1	16.7	16.8	17.4	18	18.3	18.5
Flu	L-3	IAV	26.0	24.2	25	24.9	23.1	23.1	22.2	20.6	19.2	18.7	18.9	20.4	21.8	22.4	23.1	23.3	24	23.2
Flu	L-4	IAV	20.5	20.4	21.5	20.5	19.1	19.1	18.3	16.9	15.8	15.1	Euth							
Mock	L-5	PBS	21	20.6	21	20.8	21.2	212	21.3	20.6	20.8	20.8	20.9	21.2	20.7	21	21	21.4	21.1	21.4
Mock	L-6	PBS	21.4	21.1	21.6	21.3	21.9	21.9	22	21.9	21.9	22	21.6	21.7	22	22.3	22.1	22	21.8	21.9
Mock	L-7	PBS	19.8	20.2	20.7	20.4	20.8	20.8	21.2	20.5	20.4	20.7	21.1	21.2	20.8	21.4	21.5	21.8	20.7	20.7

Weight loss values (grams) following infection with Influenza A Virus (IAV) or Mock (PBS). As per animal protocol, all mice were weighed over the first seven days following infection and each subsequent day until mice succumb to infection, regained their Day 0 starting body weight, or had weight gain on 4 consecutive days. Mice losing greater than 20% body weight were ground fed and monitored multiple times per day. Mice losing >30% body weight or determined to be moribund/unlikely to recover were immediately sacrificed using humane euthanasia protocols.
