# Supplementary material for: New Metrics for Evaluating Viral Respiratory Pathogenesis
Source: PLoS One. 2015 Jun 26;10(6):e0131451. doi: 10.1371/journal.pone.0131451 (PMC4482571; doi:10.1371/journal.pone.0131451)
Supplement: S4 Table — (PDF) [file pone.0131451.s004.pdf]

**Supporting Information Table S4. Daily means of respiratory phenotypes across two respiratory pathogens**

| Day | Treatment | Sqrt(Freq) | Log10(TVb) | Log10(MVb) | Log10(Penh) | LN(Rpef) | Sqrt(PIF) | Sqrt(PEF) | 1/Ti | Log10(Te) | Sqrt(EF50) | 1/Tr |
|---|---|---|---|---|---|---|---|---|---|---|---|---|
| 0 | Mock | 21.44 | -0.68 | 1.96 | -0.24 | 0.23 | 2.9 | 2.14 | 24.63 | -0.96 | 0.47 | 19.92 |
| | IAV | 19.98 | -0.59 | 1.99 | -0.32 | 0.21 | 3 | 2.17 | 21.93 | -0.88 | 0.48 | 16.79 |
| | SARS | 20.53 | -0.68 | 1.92 | -0.29 | 0.24 | 2.8 | 2.09 | 22.99 | -0.92 | 0.45 | 17.92 |
| 1 | Mock | 16.54 | -0.73 | 1.69 | -0.08 | 0.17 | 2.19 | 1.75 | 15.2 | -0.75 | 0.38 | 14.2 |
| | IAV | 18.86 | -0.65 | 1.87 | -0.24 | 0.21 | 2.74 | 1.99 | 20.75 | -0.83 | 0.43 | 15.89 |
| | SARS | 17.85 | -0.68 | 1.79 | -0.33 | 0.21 | 2.5 | 1.82 | 18.72 | -0.77 | 0.38 | 13.15 |
| 2 | Mock | 20.69 | -0.63 | 1.98 | -0.27 | 0.23 | 2.88 | 2.15 | 21.99 | -0.95 | 0.48 | 18.7 |
| | IAV | 17.52 | -0.64 | 1.82 | 0.01 | 0.14 | 2.67 | 2.11 | 18.71 | -0.77 | 0.43 | 16.41 |
| | SARS | 12.88 | -0.58 | 1.59 | 0.71 | 0.08 | 2.6 | 2.47 | 15.55 | -0.45 | 0.52 | 20.12 |
| 3 | Mock | 18.2 | -0.66 | 1.84 | -0.23 | 0.21 | 2.67 | 1.96 | 19.46 | -0.79 | 0.39 | 14.83 |
| | IAV | 18.12 | -0.46 | 2.02 | 0.32 | 0.12 | 3.48 | 2.93 | 21.3 | -0.77 | 0.61 | 24.2 |
| | SARS | 13.74 | -0.58 | 1.64 | 0.89 | 0.11 | 2.67 | 2.79 | 15.5 | -0.49 | 0.69 | 29.18 |
| 4 | Mock | 18.05 | -0.64 | 1.85 | -0.22 | 0.22 | 2.64 | 1.94 | 18.07 | -0.81 | 0.41 | 15.12 |
| | IAV | 18.11 | -0.49 | 2.01 | 0.21 | 0.12 | 3.38 | 2.74 | 21.19 | -0.8 | 0.14 | 15.16 |
| | SARS | 14.66 | -0.6 | 1.7 | 0.61 | 0.1 | 2.94 | 2.52 | 17.41 | -0.58 | 0.61 | 26.93 |
| 7 | Mock | 20.08 | -0.63 | 1.96 | -0.24 | 0.24 | 2.89 | 2.17 | 21.57 | -0.9 | 0.47 | 17.87 |
| | IAV | 11.79 | -0.46 | 1.65 | 1.09 | 0.05 | 3.01 | 3.08 | 16.28 | -0.37 | 0.77 | 29.16 |
| | SARS | 18.65 | -0.64 | 1.89 | 0.27 | 0.15 | 2.9 | 2.45 | 21.66 | -0.85 | 0.62 | 29.61 |
| 10 | Mock | 20.26 | -0.65 | 1.95 | -0.23 | 0.23 | 2.88 | 2.13 | 22.19 | -0.91 | 0.45 | 17.93 |
| | IAV | 19.12 | -0.53 | 2.03 | 0.45 | 0.14 | 3.34 | 2.96 | 21.99 | -0.86 | 0.74 | 33.39 |
| | SARS | 18.9 | -0.59 | 1.94 | 0.06 | 0.17 | 2.94 | 2.41 | 20.08 | -0.87 | 0.57 | 25.36 |
| 14 | Mock | 20.45 | -0.63 | 1.97 | -0.4 | 0.28 | 2.98 | 2.12 | 23.1 | -0.9 | 0.45 | 16.03 |
| | IAV | 18.61 | -0.59 | 1.94 | 0.2 | 0.15 | 2.93 | 2.57 | 19.62 | -0.84 | 0.61 | 24.95 |
| | SARS | 20.69 | -0.58 | 2.04 | 0.04 | 0.2 | 3.17 | 2.59 | 22.82 | -0.95 | 0.62 | 27.62 |
| 21 | Mock | 19.2 | -0.63 | 1.9 | -0.27 | 0.2 | 2.86 | 2.1 | 21.67 | -0.83 | 0.41 | 15.23 |
| | IAV | 19.49 | -0.57 | 1.99 | -0.14 | 0.16 | 3.01 | 2.33 | 20.78 | -0.87 | 0.47 | 18.21 |
| | SARS | 19.75 | -0.58 | 2 | -0.13 | 0.19 | 2.92 | 2.29 | 19.67 | -0.92 | 0.48 | 19.97 |
| 28 | Mock | 18.52 | -0.6 | 1.91 | -0.33 | 0.26 | 2.83 | 2.02 | 19.48 | -0.81 | 0.41 | 13.94 |
| | IAV | 18.85 | -0.56 | 1.97 | -0.23 | 0.18 | 2.99 | 2.21 | 20.23 | -0.84 | 0.43 | 15.62 |
| | SARS | 21.85 | -0.56 | 2.11 | -0.21 | 0.21 | 3.26 | 2.51 | 23.33 | -1 | 0.53 | 22.49 |
