# Supplementary material for: New Metrics for Evaluating Viral Respiratory Pathogenesis
Source: PLoS One. 2015 Jun 26;10(6):e0131451. doi: 10.1371/journal.pone.0131451 (PMC4482571; doi:10.1371/journal.pone.0131451)
Supplement: S5 Table — (PDF) [file pone.0131451.s005.pdf]

Supporting Information Table S5: Significant Differences in Respiratory Function in specific virus infection

Phenotype	Differences	Timepoints
Sqrt(Freq)	Infected vs. Mock	D2, D7
	SARS vs. Flu	D7
Log_{10}(TVb)	Infected vs. Mock	D3, D4, D7
	SARS vs. Flu	D3
Log_{10}(MVb)	Infected vs. Mock	D7
	SARS vs. Flu	N/A
Log_{10}(Penh)	Infected vs. Mock	D2-D4, D7, D10, D28
	SARS vs. Flu	D2, D7, D10
LN(Rpef)	Infected vs. Mock	D2-D4, D7, D10, D14, D21
	SARS vs. Flu	D7
Sqrt(PIF)	Infected vs. Mock	D3, D4
	SARS vs. Flu	D3, D4
Sqrt(PEF)	Infected vs. Mock	D3, D4, D7
	SARS vs. Flu	N/A
1/Ti	Infected vs. Mock	D2, D7
	SARS vs. Flu	D2, D7
Log_{10}(Te)	Infected vs. Mock	D7
	SARS vs. Flu	D7
Sqrt(EF50)	Infected vs. Mock	D3, D7
	SARS vs. Flu	N/A
1/Tr	Infected vs. Mock	D3
	SARS vs. Flu	N/A

For each phenotype at each day, a partial F-test was used to identify those days on which treatment had a significant effect on respiratory phenotypes. We then used Tukey's HSD to identify whether those significant effects of treatment were due to respiratory differences between mock and infected animals, or between the different dose categories. Phenotypes: Freq=frequency, TVb=Tidal Volume, MBv=Minute Volume, penH=Enhanced Pause, rPEF=ratio of time to peak expiratory flow, PIFb=peak inspiration, PEFb=peak expiration, Ti=inspiratory time (milliseconds), Te=expiratory time (milliseconds), EF50=Mid-tidal expiratory flow, Tr=relaxation time
