# Supplementary material for: New Metrics for Evaluating Viral Respiratory Pathogenesis
Source: PLoS One. 2015 Jun 26;10(6):e0131451. doi: 10.1371/journal.pone.0131451 (PMC4482571; doi:10.1371/journal.pone.0131451)
Supplement: S6 Table — (PDF) [file pone.0131451.s006.pdf]

Supporting Information Table S6. Histopathology Scoring of Lung Disease

	SARS			IAV-H1N1-09		
	D2	D4	D7	D2	D4	D7
Airway (Denudation, Debris, Inflammation, Necrosis)	2.2	1.3	2	0.25	3.75	2.13
Airway Debris	1	0.5	0.5	0.25	1.5	0.81
Vasculature (Perivascular Cuffing, Edema)	1.4	1.6	2.1	0	2	2
Alveoli/Parenchyma (Interstitial Septum, Airspace Inflammation)	0.6	1.2	2.6	0	1.13	1.5

Histopathological scoring is based on blinded scoring of between 2-5 animals per virus and timepoint. Scores range from 0-3 (none, mild, moderate or severe). Lung sections are from animals used in [15, 27].
